# Allometry and Distribution of Nitrogen in Natural Plant Communities of the Tibetan Plateau

**DOI:** 10.3389/fpls.2022.845813

**Published:** 2022-03-11

**Authors:** Xin Li, Mingxu Li, Li Xu, Congcong Liu, Wenzong Zhao, Changjin Cheng, Nianpeng He

**Affiliations:** ^1^Key Laboratory of Ecosystem Network Observation and Modeling, Institute of Geographic Sciences and Natural Resources Research, Chinese Academy of Sciences, Beijing, China; ^2^College of Resources and Environment, University of Chinese Academy of Sciences, Beijing, China; ^3^College of Life and Environmental Sciences, Minzu University of China, Beijing, China; ^4^School of Ecology and Nature Conservation, Beijing Forestry University, Beijing, China; ^5^Center for Ecological Research, Northeast Forestry University, Harbin, China

**Keywords:** nitrogen, allometry, environmental regulation, homeostasis, plant functional group

## Abstract

Nitrogen (N) is an important element for most terrestrial ecosystems; its variation among different plant organs, and allocation mechanisms are the basis for the structural stability and functional optimization of natural plant communities. The nature of spatial variations of N and its allocation mechanisms in plants in the Tibetan Plateau—known as the world’s third pole—have not been reported on a large scale. In this study, we consistently investigated the N content in different organs of plants in 1564 natural community plots in Tibet Plateau, using a standard spatial-grid sampling setup. On average, the N content was estimated to be 19.21, 4.12, 1.14, and 10.86 mg g^–1^ in the leaf, branch, trunk, and root, respectively, with small spatial variations. Among organs in communities, leaves were the most active, and had the highest N content, independent of the spatial location; as for vegetation type, communities dominated by herbaceous plants had higher N content than those dominated by woody plants. Furthermore, the allocation of N among different plant organs was allometric, and not significantly influenced by vegetation types and environmental factors; the homeostasis of N was also not affected much by the environment, and varied among the plant organs. In addition, the N allocation strategy within Tibet Plateau for different plant organs was observed to be consistent with that in China. Our findings systematically explore for the first time, the spatial variations in N and allometric mechanisms in natural plant communities in Tibet Plateau and establish a spatial-parameters database to optimize N cycle models.

## Introduction

Nitrogen (N) is an essential nutrient for plant growth and survival, and is also an important element limiting productivity in most terrestrial ecosystems (Xu et al., 2020; Xu and He, 2020). How to allocate N among different plant organs is as fundamental as resource acquisition by the plant, affecting material and energy cycles in ecosystems. Although there are many studies on intra- and interspecific variability in N allocation at species-level, it is difficult to establish a direct link between these studies and the ecosystem function, due to scale mismatch, as discussed by He et al. (2019). The N content at community-level, on the other hand, can be combined with remote sensing and ecological models at regional scales (Zhang et al., 2018; He et al., 2020).

N is associated with the rate of photosynthesis, and total carbohydrate storage in leaves, branch, trunk, and roots (Wang et al., 2020). The allocation of N among plant organs undoubtedly reflects the adaptation strategies of natural communities at a large scale. To maximize organ function, one of the nutrient allocation strategies adopted by plants is that more active organs are allocated more nutrients (Zhang et al., 2018); it is therefore presumed that in terms of N content, the leaves and roots contain higher N than the branches and stems. Adjusting N allocation rates among different organs is also an important way for plants to adapt to their environment. Usually, plants can selectively absorb and accumulate nutrients to maintain intracellular elemental balance and achieve optimal function; that is, the ratios of various elements in the cells are relatively stable during biochemical processes (Hermans et al., 2006; Cleveland and Liptzin, 2007). It has been demonstrated that the pattern of allocation of multiple nutrients among major plant organs is conserved (Zhao et al., 2016). However, most of the above studies on N content and distribution are at the species scale, on the basis of the biochemical conversion of N during the protein synthesis process, it is speculated that the interrelation among N allocation to different organs in community may be similarly conserved to some extent.

The Tibetan Plateau—considered the world’s third pole—is the best natural laboratory for the study of nutrient allocation in natural plant communities (Siwen, 1997), especially under the scenario of global climate change. Here, available soil N is only about 1% due to the low temperatures, and plant growth may be subjected to greater limitations due to lower N availability (Kou et al., 2020). Therefore, it is essential to address the spatial variation and allocation mechanisms of N among different plant organs in the natural plant communities on Tibet Plateau. Although many factors may regulate the spatial variation of N—such as precipitation, temperature, CO_2_, O_2_, light, and other environmental factors (Han et al., 2011; Wang et al., 2014; De Frenne et al., 2018)—plants can prevent the internal concentration of N from changing drastically in response to external environmental changes, through their own regulatory mechanisms such as homeostasis (Yu et al., 2011). To date, it is unclear how the N homeostasis in different organs of plants is maintained in natural communities, when considered at a large scale.

**FIGURE 1:**
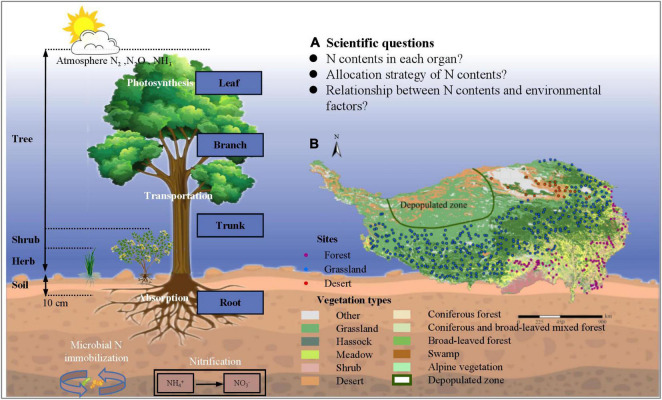
Theoretical framework for allometry of nitrogen allocation in natural plant communities of Tibetan Plateau. **(A)** Overview of scientific questions of our research; **(B)** Spatial distribution of sampling plots on Tibetan Plateau. 1564 plots were chosen to conduct field investigation; areas such as depopulated parts of Tibet were not sampled due to concerns about traffic, climate, and safety of researchers.

In this study, we systematically investigated the N content of different plant organs (leaf, branch, trunk, and root) in 1564 natural plant communities on Tibet Plateau through a standardized spatial-grid sampling method, to demonstrate the spatial variation of N among different plant organs, as well as plant allometry, in the natural plant communities on Tibet Plateau. The specific objectives are to quantify the spatial variation of N among different organs of plant communities in Tibet Plateau, and to explore N partitioning relationships and potential mechanisms that act on a large scale (Figure 1). Furthermore, we hope to test three hypotheses in relation to Tibet Plateau; (1) accumulation of N content of community level is higher in more active organs (2) the pattern of N allocation in plant community is conserved (3) Although plants on Tibet Plateau are subject to a variety of environmental stresses, N in different organs of plant community has high level homeostasis.

## Materials and Methods

### Study Sites

Tibet Plateau (26°00′–39°47′ N, 73°19′–104°47′ E) (Figure 1B), the average altitude is over 4000 meters, and the average annual temperature in the plateau hinterland is below 0°C, is a giant tectonic geomorphic unit, and the highest plateau in the world. It has a unique natural environment and spatial differentiation pattern, constrained by reduced atmospheric circulation and the distinct topography of the plateau with a unique geographical combination of water and heat conditions. Furthermore, it is considered a sensitive area with reference to global climate change, and the largest ecologically fragile area in the world.

### Field Sampling

Field surveys and sampling were conducted from 2019 to 2021 during the peak period for plant growth (July and August). A total of 1564 natural communities were investigated, three plots for each community, using a rasterized sampling method with a grid size of 0.5° in the latitudinal and longitudinal directions. In practice, some designated plots were not investigated, especially in the depopulated zones of Tibet Plateau, due to concerns of traffic, climate, and researcher safety. Within each grid, we selected natural plant communities with less human influence as the sample plots, and recorded their geographical information such as latitude, longitude, and elevation. Uniform protocols were used for the field-sampled plants and soil as described by Zhang et al. (2018). For woody plants from forest plots, healthy and mature trees with uniform growth were selected in each plot; mature leaves on branches exposed to the sun, of three dominant plant species, were selected, as were healthy and mature tree branches, as samples. All root samples were obtained by drilling using a 9 cm diameter soil auger at 0–30 cm depth, and the trunk was drilled with a growth cone to obtain the sample; these samples of individual sub-organs from multiple species were mixed and brought back to the laboratory for subsequent processing. For herbaceous plants, 1 m × 1 m were set up and the entire part of the plant above the ground were collected from the field, and root samples from the 0 to 30 cm soil layer were collected using a 9 cm diameter soil auger. All plant samples were mixed, that is, all plants from the same organ were mixed to obtain a mixed sample, use the average of multiple species, the specific calculation method of nitrogen content in community as follows.


$$N_{C\,o\,m,i}=\frac{\sum N_{S\,p\,e,i}}{n}$$

where *N_*Com*_ and N_*Spe*_* represent the N content (mg g^–1^) of plant community and species, respectively, *i* is the specific plant organs (such as leaf, branch, trunk, or root). *n* is the sample number of species taken.

Similarly soil samples were collected using the soil auger from each plot, after removing weeds and surface litter (Zhao et al., 2016).

### Measurement of Nitrogen Content

First, the fresh plant samples were dried in an oven at 60–70°C to remove the water gradually (Zhang et al., 2018). Plant roots and gravel were manually removed from the soil samples, sieved through a 2 mm sieve, and dried naturally. Then, all plant and soil samples were ground to a powder using an agate mortar (RM200, Retsch, Haan, Germany) and a ball mill (RM200; Retsch, Haan, Germany). Finally, the N content of all samples was measured using an elemental analyzer (Vario MAX CN Elemental Analyzer, Elementar, Germany). pH of the soils was determined using a pH meter (Ultrameter II, United States) (Wang et al., 2021).

### Environmental Variables

Based on the information on the location of each site, we extracted data on total solar radiation (TSR, mol m^–2^ d^–1^), Mean annual precipitation temperature (MAT, °C), growing season mean temperature (GST, °C), coldest month mean temperature (*T*_*Coldest*_, °C), warmest month mean temperature (T_*Warmest*_, °C), Mean annual precipitation (MAP, mm), growing season precipitation (GSP, mm), wettest month precipitation (P_*Wettest*_, mm) and driest month precipitation (P_*Driest*_, mm) from was obtained from Worldclim Database^1^ ; From the Science Data Bank,^2^ we extracted photosynthetic active radiation (PAR, mol m^–2^ d^–1^) and ultraviolet radiation (UR, MJ m^–2^ d^–1^) data for 2000–2014 (Li et al., 2021).

Based on the altitude, and using the method proposed by Kouwenberg et al. (2007), the oxygen partial pressure (*PO*_2_) was calculated, as given below.


$$P\,C\,O_{2}=\left[\frac{P_{a\,i\,r,z}}{101325}\right]*P\,C\,O_{2,s\,e\,a-l\,e\,v\,e\,l}$$

where *PO*_2_ (Pa) is calculated from altitude (z, in m) and mean July temperature (T, °C); T is calculated considering a lapse rate of 0.6 °C per 100 m of altitude. *P*CO_2,sea–level_ is the CO_2_ partial pressure at sea-level.

### Data Analysis

N content was calculated and analyzed using Excel 2010 and SPSS21.0 (Chicago, IL, United States) software, and the N content of different organs, communities, vegetation types and eco--geographical areas were compared using the one-way ANOVA with multiple comparisons (LSD) method. The relationships between N content and environmental factors were analyzed by the randomForest model, using the randomForest^3^ and rfPermute^4^ packages in R 3.5.3 (Jiao et al., 2018), and the correlations between N content and environmental variables were characterized by Pearson correlation coefficients, which were plotted in Origin 2019 and R software (version 3.5.1, Development Core Team, Vienna, Austria).

In addition, we constructed Structural Equation Models (SEMs) to explore the direct and indirect effects of various factors on the N content of different organs. Considering the covariate effects among the different variables, the variables were classified into four major groups based on the results of correlation analysis, temperature, precipitation, radiation, soil and PO_2_, and each major group was analyzed using Principal Component Analysis (PCA) in order to construct a composite indicator to characterize the information regarding each variable. Within each group, only variables with a significant correlation with N content were included in the PCA. In the SEM analysis, the data were fitted using the Maximum Likelihood Estimate; model fit was expressed using the following metrics: *p* > 0.05, ratio of cube to degrees of freedom (χ2/df) in the range 1–3, model fit (GIF) > 0.9, and root mean square error (RMSEA) < 0.08 (IBM, Chicago, IL, United States) (Wang et al., 2017).

The equation for allocation of N among plant organs is given below Eq. (1) (Reich et al., 2010).


(1) l⁢o⁢g⁢y=b⁢l⁢o⁢g⁢x+l⁢o⁢g⁢a

where y and x represent the N content (mg g^–1^) of two specific plant organs (such as leaf, branch, trunk, or root). a, of the fitted line, b, the anisotropic partitioning index which can reflect the allometric allocation rate of N among different organs is represented by the slope, when b = 1, it means that the distribution relationship is isometric, otherwise it is allometric, when the slope is greater than 1, it is considered that the change rate of y is greater than x, otherwise, the change rate of x is greater than y Standardized major axis regression (SMA) was used to analyze the allometry with respect to N, among plant organs (Sokal and Rohlf, 2013); the “lmodel2” function in the “lmodel2” package in R was used for the fitting of various parameters in SMA. The likelihood ratio test was used to test whether there were significant differences in the allometric growth index (*slope*) among different organs. This test was performed using the “smart” function of the “smart” package in R (Zhao et al., 2016).

Sterner and Elser (2002) proposed an internal stability model which expressed the relationship between the stoichiometric characteristics of organisms and environmental stoichiometric characteristics, as follows;


(2) $$\frac{d\,y}{y}=\frac{1}{H}\,\frac{d\,x}{x}$$

here, H is the homeostasis index; x is the supply of nutrients in the environment, that is, the N content of the soil considered in this study; y is the N content of organisms. In this study, N with subscripts refer to the N content in the corresponding organs; x and y are expressed as a percentage of the element or as the proportion of the element, in the organ, indicated as n% or *N*_*leaf*_: *N*_*root*_, etc. Expressing the formula in exponential form as in Eq. (3) below,


(3) $$y=c\,x^{\frac{1}{H}}$$

where c is a constant. An absolute value of *H* that is greater than one is considered to maintain homeostasis (Sterner and Elser, 2002). The value of 1/H (0 < 1/H < 1) is often used as a measure of the strength of endostatins. 1/H is classified into four categories: 0 < 1/H < 0.25, essentially stable, i.e., high level of homeostasis; 0.25 < 1/H < 0.5, weakly stable; 0.5 < 1/H < 0.75, weakly sensitive; and 1/H > 0.75, sensitive, i.e., essentially unstable. When the results of model simulation are not significant, an absolute steady state is indicated (Yu et al., 2015).

## Results

### Variation of Nitrogen Content Among Plant Organs

**FIGURE 2:**
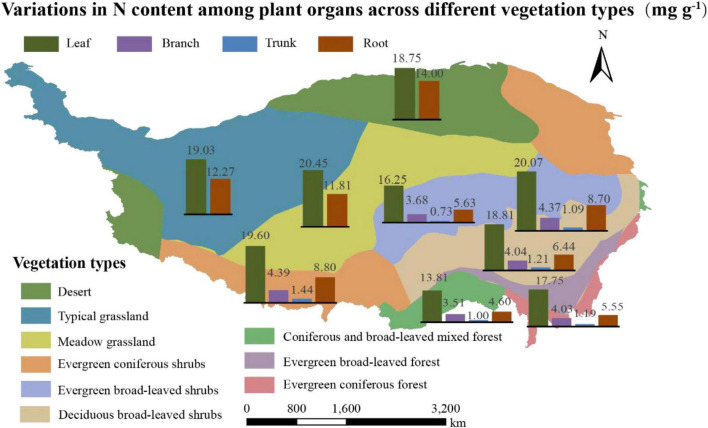
Variations in N content (mg g^– 1^) among plant organs across different vegetation types. Vegetation zoning is based on dominant vegetation type with reference to vegetation types found in China.

The N content of the organs, considering the average of 1564 communities on Tibet Plateau, was 19.21 mg g^–1^ for leaves (*N*_*leaf*_), 4.11 mg g^–1^ for branches (*N*_*branch*_), 1.14 mg g^–1^ for trunks (*N*_*trunk*_), and 10.86 mg g^–1^ for roots (*N*_*root*_); the N content thus showed significant variation among the different organs (*p* < 0.01) (Supplementary Table 1). In terms of different plant communities, the ones in the desert had the highest *N*_*root*_ and those in the forests had the lowest *N*_*root*_ (*p* < 0.01) (Table 1). The N content was also significantly different across vegetation types on a spatial scale (Figure 2 and Supplementary Table 2); in communities where herbaceous plants dominated, *N*_*root*_ values were generally higher compared to tree-dominated communities.

**TABLE 1:** Changes in nitrogen content (N, mg g^–1^) of plant organs in natural plant communities of forests, grasslands, and deserts.

Organs	Types	No.	Mean (mg g^–1^)	Min (mg g^–1^)	Max (mg g^–1^)	SE	Skewness	Kurtosis	CV (%)
Leaf	Forest	120	18.6	8.25	32.89	0.44	0.44	0.12	0.26
	Grassland	367	19.46	5.59	32.45	0.24	–0.20	0.43	0.23
	Desert	23	18.57	11.50	28.45	0.99	0.47	–0.69	0.27
	Total	510	19.21	5.59	32.89	0.20	–0.02	0.18	0.24
Branch	Forest	104	4.12	1.50	8.80	0.14	0.55	0.55	0.35
Trunk	Forest	86	1.14	0.50	1.98	0.03	0.30	–0.35	0.28
Root	Forest	120	6.11*^c^***^†^**	1.10	16.16	0.28	1.02	1.07	0.49
	Grassland	375	12.21*^b^*	4.50	24.06	0.19	0.26	–0.06	0.29
	Desert	22	13.88*^a^*	6.43	22.82	0.93	0.35	–0.45	0.31
	Total	517	10.86	1.10	24.06	0.19	0.11	–0.40	0.40

***^†^** Different letters indicate significant differences at the p = 0.05 level.*

### Nitrogen Allocation Among Various Plant Organs

According to the allocation index for each organ (Table 2), the rate of change of N in each organ was different—in the order *N*_*leaf*_ > *N_*root*_* > *N*_*branch*_ > *N*_*trunk*_; the N content between different organs showed a significant positive correlation (*p* < 0.01). In general, N allocation in leaves and roots was isometric (no significant difference between the slope of equation and 1 at *p* = 0.05), while in other organs, it was allometric (Table 2). Further investigation of N allocation in leaves and roots revealed that it was only isometric in deserts, whereas forests and grasslands showed a significant allometric N allocation (*p* < 0.001; Supplementary Table 4).

**TABLE 2:** Key parameters of allometric equations for N in plant communities of Tibetan Plateau.

Organs (x vs. y)	n	R^2†^	*p*	Slope	95%CI	Intercept	95%CI	
Leaf vs. Branch	98	0.52	< 0.001	0.30	(0.26, 0.35)	–1.54	(–2.36, –0.72)	*** ^‡^
Leaf vs. Trunk	80	0.11	0.002	0.07	(0.06, 0.09)	–0.12	(–0.41, 0.16)	***
Leaf vs. Root	503	0.04	< 0.001	0.96	(0.88, 1.04)	–7.39	(–9.04, –5.75)	ns
Branch vs. Trunk	84	0.14	< 0.001	0.26	(90.22, 0.32)	0.15	(–0.07, 0.36)	***
Branch vs. Root	101	0.22	< 0.001	1.69	(91.42, 2.01)	–1.47	(–2.79, –0.16)	***
Trunk vs. Root	85	0.09	0.006	6.48	(5.27,7.97)	–2.18	(–3.81, –0.55)	***

*^†^ R^2^, the coefficient of determination; CI, confidence interval.*

^‡^ ***, denote significant differences between the slope of equation and 1 at p = 0.001; ns, no significant difference between the slope of equation and 1 at p = 0.05. Note that nitrogen contents of all plant organs were transformed into a log–log scale. The likelihood ratio test was used to examine differences among different organs; The data were analyzed by standardized major axis (SMA) and calculated by SMATR Version 2.0 (http://www.bio.mq.edu.au/ecology/SMATR/).

### Influence of Environmental Factors on Nitrogen Variation and Allocation

**FIGURE 3:**
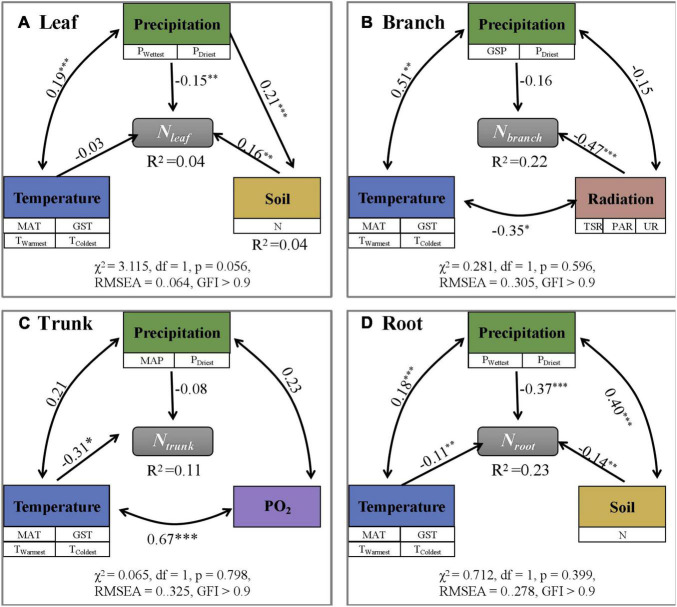
Path analysis exploring direct and indirect effects of environmental factors on N content (mg g^– 1^) in different plant organs (**A–D**: Leaf, Branch, Trunk, and Root). N content is influenced by a variety of environmental factors, but the overall revelation is low (*R*^2^ < 0.25). The symbols ***, **, and * represent *p* < 0.001, *p* < 0.01, and *p* < 0.05, separately.

Among different eco-geographical areas (Supplementary Table 3), *N*_*leaf*_ was the lowest and *N*_*root*_ was the highest in the humid area of the sub-frigid zone in the subtropical humid area, while *N*_*branch*_, and *N*_*trunk*_ values were lowest in humid area of the subtropical zone. In other words, the N content in different plant organs did not show a consistent change with increasing or decreasing temperature and moisture on Tibet Plateau. Analyses using SEM (Figure 3 and Supplementary Figure 1) and randomForest (Supplementary Figure 2) models showed that the N content of individual organs were directly or indirectly influenced by different environmental factors. However, considering the low degree of interpretation (SEM; *R*^2^≤ 0.23, randomForest; *R*^2^ ≤ 0.38), they were not sufficient to establish that the environment had a driving role in deciding the N content.

Considering the coefficients of variation (CV) (Table 1 and Supplementary Tables 1–3), all values of CV were less than or equal to 0.50, indicating relative stability and low plasticity. From Supplementary Figure 4, the absolute value of 1 / H for *N*_*leaf*_, *N*_*branch*_, *N*_*trunk*_, and *N*_*root*_ are 0.03, 0.12, 0.01, and 0.09, respectively, and all of them are less than 0.25, indicating high homeostasis.

## Discussion

### Spatial Variation and Allocation of Nitrogen Among Different Organs in Plant Communities of Tibetan Plateau

The study explores for the first time, the spatial variation and allocation of N among different plant organs, in a unified and standardized field investigation in Tibet Plateau, It is an important research field of ecology to reveal the strategies of plants to the environment by exploring the spatial variation of N, a large number of studies have shown that plant traits vary greatly in different vegetation floras and at global scales, mainly due to the combined effects of intraspecific variation, climatic factors and soil environmental factors(Han et al., 2011; Liu et al., 2013). Compared with previous studies, the *N*_*leaf*_ (19.21 mg g^–1^) was observed to be lower than the world average (19.3 mg g^–1^) (Wright et al., 2004) and the average value for China (20.5 mg g^–1^) (Zhao et al., 2019). However, the average value for *N*_*root*_ (10.86 mg g^–1^) was higher than in China (9.20 mg g^–1^) (Zhao et al., 2019); the different methods adopted by different researchers, and the incompatibility between the organs sampled at different sampling sites (Zhang et al., 2018) may have been the reasons for the non-uniform results. Previous researches on N in Tibet Plateau ignored the role of the community-effect in nature, focusing instead on experiments on individual species under controlled conditions (Zhao et al., 2020). The few studies carried out on N allocation in plants on Tibet Plateau were also limited to specific organs such as the leaf or root, at specific sites; considering studies worldwide, most studies on N allocation at a regional scale mainly focused on the leaf and root (Geng et al., 2014). Through exploring the N allocation in plant communities using actual measurement data, we can better understand the conditions related to nutrient limitation, and establish a theoretical basis for the sustainable management of natural ecosystems such as the alpine ecosystems. Good models are often based on and verified by measured data; therefore, this systematically collated information on N allocation for different vegetation types, at a large scale, may prove important for the optimization of N cycle models for unique geographical regions.

### Presence of Higher Nitrogen Content in Active Organs With Faster Growth Rate

Insufficient N is a common limiting factor for the growth of individual plants and even natural plant communities (Stevens et al., 2004). Leaves, as the most active organ, require large amounts of N to support the carbon fixation phase of photosynthesis involving light capture, and influences photosynthesis rates as well as plant productivity (Manzoni et al., 2008). Roots can absorb inorganic salts and minerals from the soils; roots are sensitive to changing environments, and cooperate with the plant organs above ground to optimize plant growth (Dinneny et al., 2008). Compared with the storage organs or supporting organs such as branches and trunks, the root has a greater role to play in plant growth. Therefore, *N*_*root*_ is significantly higher than *N*_*branch*_ and *N*_*trunk*_ (Supplementary Table 1), and the rates of change are such that *N*_*leaf*_ > *N*_*root*_ > *N*_*branch*_ > *N*_*trunk*_ (Table 2). These results verified our first hypothesis that the more active organs have more N content. However, some studies have shown no significant difference between *N*_*branch*_ and *N*_*trunk*_, possibly because the amount of an element present in an organ is related to the age of the organ (Kramer and Kozlowski, 1979). Furthermore, differences in sampling and analysis from the perspective of the plant community as a whole, may result in such errors.

**FIGURE 4:**
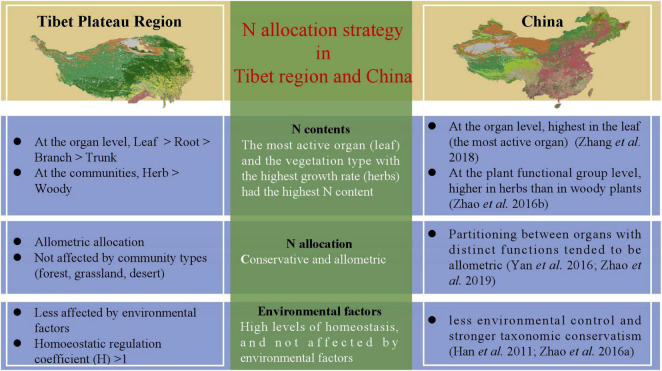
Strategies for nitrogen allocation are uniform across Tibetan Plateau region and the whole of China. Nitrogen allocation strategy in Tibetan Plateau is consistent with that in the whole of China.

There were significant differences in the *N*_*root*_ of different vegetation types (Figure 2 and Supplementary Table 2); communities dominated by woody vegetation had lower N content than communities dominated by herbaceous plants. According to the growth rate hypothesis (Ågren, 2004), herbaceous plants are shorter-lived and have higher growth rates, so they require a root system with a higher rate of uptake to match a faster growth rate. In addition, it is possible that there is more lignified tissue in woody plants, diluting the N content (Kerkhoff et al., 2006). The *N*_*root*_ varied significantly among different types of plant communities, with plant communities in deserts exhibiting the highest *N*_*root*_ (Table 1). One explanation for the higher *N*_*root*_ in deserts is that higher levels of NH_4_^+^ can promote root uptake under drought conditions, and thus increase resistance to drought stress (Gao et al., 2009). Another possible reason for the higher proportion of N absorption in the root in deserts is for the root system to store more nutrients so that the water absorption capacity of plants is enhanced to ensure their survival. At the same time, these results also verified our hypothesis that higher the activity, higher the N content; this hypothesis has also been verified in a study of the N allocation strategies in plant communities in China (Zhao et al., 2016; Zhang et al., 2018) (Figure 4).

### Conservative Anisotropic Allocation Among Different Organs in Natural Plant Communities

The strategy adopted for allocating limited resources is critical for plants to adapt to the environment (Chapin et al., 1987). The law of allometric allocation was proposed by Huxley in 1924 which states that the growth rates of different parts of an organism are not equal (Vretare et al., 2001). It was argued that each individual plant has a certain allometric growth pattern under certain conditions, which may be influenced by changing growth rates and shapes (Lu et al., 2007). The results of our study on N allocation in natural plant communities showed that N is mostly allocated allometrically in different organs (Table 2), but isometric allocation between *N*_*leaf*_ and *N*_*root*_ in deserts has been observed (Supplementary Table 4). These phenomena may have been exhibited only by a small number of plant species at a particular site, while there could be large variation among species; therefore, the results from a local site may not represent well, the region as a whole (Zhang et al., 2018). Each organ is able to absorb a unique share of the material nutrients available (Lu et al., 2007), and therefore grows at a certain rate; allometry among different organs may thus reflect the allocation process leading to a steady state. Some studies on N-P allocation have demonstrated that leaves and propagules have more conservative nutrient allocation than stems and underground organs (Minden and Kleyer, 2014). Through separately exploring N allocation relationships, we found that the large variability in the data did not result in significant differences in allometric allocation among different plant communities, except *N*_*leaf*_ and *N*_*root*_ allocations in deserts. In other words, N allocation was generally unaffected by the type of plant community, and the allocation relationships were conservative and allometric. Compared to the study of N allocation in woody plants by Yan et al. (2016), and previous studies on Nallocation supported allometric allocation which was conservative at a spatial level (Figure 4).

### Homeostatic Regulation of Nitrogen in Plant Communities and Low Influence of Environmental Factors

The climate and soil conditions in TP are relatively unsuitable for plant growth due to the high altitude; at high altitudes, the nutrient turnover rate decreases and the nutrient cycle process slows down (Reich and Oleksyn, 2004). Plants have evolved unique survival mechanisms to adapt to distinct habitats with special characteristics such as high altitude and low temperature as in TP (Wang et al., 2020). Climate change including global warming and increasing snow depth significantly affected *N*_*leaf*_ in the tundra (Welker et al., 2005) while enhanced radiation significantly increases the *N*_*leaf*_ partition coefficient (Sullivan et al., 2003). The results of N addition experiments in TP showed that N enrichment promoted plant growth, confirming that plant growth was limited by the available N in the soil (Song et al., 2012). However, there was no evidence to prove that changes in environmental factors such as nitrogen deposition have significant effects on the N content in different organs of plants on Tibet Plateau (Zhao et al., 2010). Through SEM (Figure 3 and Supplementary Figure 1) and randomForest (Supplementary Figure 2), we found that the N content in all organs is negatively affected by precipitation. It may be that, while the increase in precipitation accelerates the part of the N cycle in the ecosystem between plants and the soil—resulting in faster decomposition of litter (Austin and Sala, 2002)—the impact of increased precipitation on vegetation growth is greater than that on soil N mineralization so that increased precipitation ultimately results in dilution of N in plants (Barrett et al., 2002). Under drought conditions, plants may enhance *N*_*leaf*_ allocation so as to improve the osmotic pressure inside the cells and reduce water loss from the body. Lower R^2^ values confirmed that environmental factors are not the main drivers of N variation among different organs of the individuals in the natural plant community; the variation in N content may be due to the differences in species composition on a large scale (Han et al., 2011).

Looking at the coefficient of variation (CV), it is seen that CV is less than 50% (Table 1) for all the samples, indicating that the plasticity of N content in each organ of the community, as well as its sensitivity to the external environment, is low. For a healthy plant, there is a certain amount of homeostasis (It refers to the coordination of various organs and systems through the regulation of normal plants to maintain the relative stability of the internal environment), and it is also the condition for the healthy survival of organisms. The results in Supplementary Figure 4 show that the absolute value of the homoeostatic regulation coefficient (H) of plant organs is greater than one, which is consistent with results of previous studies (Schreeg et al., 2014). Some studies have reported that the level of homeostasis of leaves is higher than that of roots, which is consistent with recent studies (Garrish et al., 2010), indicating that leaves have a stronger ability to regulate internal N than roots. In long-term evolution of organisms, plants adapt to environmental changes by maintaining a relatively stable chemical composition of the plant body (Elser et al., 2010), whereas nutrient utilization by plants with strong level homeostasis and more conservative (Figure 4).

## Conclusion

We systematically measured the N content of different plant organs using a spatial-grid sampling method, and comprehensively explored the spatial variation of N in plants of the natural plant communities of Tibet Plateau. The results show that herbaceous plants with higher growth rates and more actively growing leaves and roots tend to accumulate more N. Being a conservative strategy, allometric distribution does not vary among different communities. The N contents in different organs exhibit homeostasis, and are not apparently influenced by environmental factors. In general, the allocation of N among different plant organs in the natural plant community is dependent on organ activity, plant growth rate, environmental factors. The N allocation strategy in the plant community in Tibet Plateau are consistent with those in China. Our results are instructive for the study of N characteristics of alpine ecosystems and global N balance. More importantly, the spatial variation patterns of N among different plant organs are key to improving the fitting accuracy of regional N cycle models.

## Data Availability Statement

The original contributions presented in the study are included in the article/Supplementary Material, further inquiries can be directed to the corresponding authors.

## Author Contributions

NH and ML designed the research. XL, LX, CL, WZ, and CC conducted the fieldwork and collected the data. XL and NH led the writing of the manuscript. All authors contributed critically to the drafts and gave final approval for publication.

## Conflict of Interest

The authors declare that the research was conducted in the absence of any commercial or financial relationships that could be construed as a potential conflict of interest.

## Publisher’s Note

All claims expressed in this article are solely those of the authors and do not necessarily represent those of their affiliated organizations, or those of the publisher, the editors and the reviewers. Any product that may be evaluated in this article, or claim that may be made by its manufacturer, is not guaranteed or endorsed by the publisher.

## Footnotes

1
http://worldclim.org/version2

2
http://www.dx.doi.org/10.11922/sciencedb.332

3
https://www.stat.berkeley.edu/~breiman/RandomForests

4
https://github.com/EricArcher/rfPermute

## Abbreviations

*N*: Nitrogen
*N*_*leaf*_: Leaf nitrogen contents (mg g^–1^)
*N*_*branch*_: Branch nitrogen contents (mg g^–1^)
*N*_*trunk*_: Trunk nitrogen contents (mg g^–1^)
*N*_*root*_: Root nitrogen contents (mg g^–1^)
*H*: Homoeostatic regulation coefficient
CV: Coefficient of variation
SEM: Structural Equation Model
TSR: Total solar radiation (mol m^–2^ d^–1^)
PAR: Photosynthetic active radiation (mol m^–2^ d^–1^)
UR: Ultraviolet radiation (MJ m^–2^ d^–1^)
MAT: Mean annual temperature (°C)
GST: Growing season mean temperature (°C)
*T*_*Coldest*_: Coldest month mean temperature (°C)
*T*_*Warmest*_: Warmest month mean temperature (°C)
MAP: Mean annual precipitation (mm)
GSP: Growing season precipitation (mm)
*P*_*Wettest*_: Wettest month precipitation (mm)
*P*_*Driest*_: Driest month precipitation (mm)
*P*O_2_: the oxygen partial pressure (Pa).
