# Supplementary material for: Allometry and Distribution of Nitrogen in Natural Plant Communities of the Tibetan Plateau
Source: Front Plant Sci. 2022 Mar 11;13:845813. doi: 10.3389/fpls.2022.845813 (PMC8963499; doi:10.3389/fpls.2022.845813)
Supplement: Supplementary file 1 [file Data_Sheet_1.docx]

**Table S1** Changes in nitrogen content (N, mg g^–1^) of individual organs among different communities in Tibet Plateau

|  | **Organs** | **No.** | **Mean**  **(mg g^–1^)** | **Min**  **(mg g^–1^)** | **Max**  **(mg g^–1^)** | **SE** | **Skewness** | **Kurtosis** | **CV** |
| --- | --- | --- | --- | --- | --- | --- | --- | --- | --- |
| Forest | leaf | 120 | 18.60^a^**^†^** | 8.25 | 32.89 | 0.44 | 0.44 | 0.12 | 0.26 |
|  | branch | 104 | 4.12^d^ | 1.50 | 8.80 | 0.14 | 0.55 | 0.55 | 0.35 |
|  | trunk | 86 | 1.14^e^ | 0.50 | 1.98 | 0.03 | 0.30 | –0.35 | 0.28 |
|  | root | 120 | 6.11^c^ | 1.10 | 16.16 | 0.28 | 1.02 | 1.07 | 0.49 |
| Grassland | leaf | 367 | 19.46 | 5.59 | 32.45 | 0.24 | –0.20 | 0.43 | 0.23 |
|  | root | 375 | 12.21 | 4.50 | 24.06 | 0.19 | 0.26 | –0.06 | 0.29 |
| Desert | leaf | 23 | 18.57 | 11.50 | 28.45 | 0.99 | 0.47 | –0.69 | 0.27 |
|  | root | 22 | 13.88 | 6.43 | 22.82 | 0.93 | 0.35 | –0.45 | 0.31 |
| Total | leaf | 510 | 19.21^b^ | 5.59 | 32.89 | 0.20 | –0.02 | 0.18 | 0.24 |
|  | branch | 104 | 4.11^c^ | 1.50 | 8.80 | 0.14 | 0.55 | 0.55 | 0.36 |
|  | trunk | 86 | 1.14^d^ | 0.50 | 1.98 | 0.03 | 0.30 | –0.35 | 0.28 |
|  | root | 517 | 10.86^a^ | 1.10 | 24.06 | 0.19 | 0.11 | –0.40 | 0.40 |

**^†^** Different letters indicate significant differences at the *p* = 0.05 level.

**Table S2** Changes in the nitrogen content (N, mg g^–1^) of plant organs among different vegetation sub–types

| **Type** | **Sub–types** | **Organs** | **Mean** (mg g^–1^) | **SE** | **Skewness** | **Kurtosis** | **Min** (mg g**^–1^**) | **Max** (mg g**^–1^**) | **CV** |
| --- | --- | --- | --- | --- | --- | --- | --- | --- | --- |
| Grassland | Desert (n = 69) | Leaf | 18.75 | 0.97 | 0.36 | –0.80 | 11.50 | 28.45 | 0.25 |
|  |  | Root | 14.00 | 0.90 | 0.27 | –0.46 | 6.43 | 22.82 | 0.31 |
|  | Meadow grassland (n = 291) | Leaf | 20.45 | 0.40 | 0.39 | 0.75 | 11.73 | 32.38 | 0.19 |
|  |  | Root | 11.81 | 0.36 | 0.14 | 0.32 | 2.50 | 22.49 | 0.30 |
|  | Typical grassland (n = 834) | Leaf | 19.03 | 0.30 | –0.22 | 0.20 | 5.59 | 32.45 | 0.25 |
|  |  | Root | 12.27 | 0.23 | 0.30 | –0.10 | 4.50 | 24.06 | 0.30 |
| Shrub | Evergreen broad–leaved shrubs (n = 53) | Leaf | 16.25^d^ | 1.66 | 1.15 | 0.53 | 10.94 | 27.81 | 0.34 |
|  |  | Branch | 3.68^b^ | 0.58 | 0.78 | –0.97 | 1.90 | 6.80 | 0.50 |
|  |  | Trunk | 0.73^a^ | 0.05 | –0.74 | 1.49 | 0.50 | 0.90 | 0.17 |
|  |  | Root | 5.63^c^ | 1.48 | 1.58 | 2.42 | 1.10 | 15.39 | 0.39 |
|  | Deciduous broad–leaved shrubs (n = 199) | Leaf | 20.07^d^ | 0.66 | 0.39 | –0.10 | 11.40 | 32.89 | 0.25 |
|  |  | Branch | 4.37^b^ | 0.32 | 0.84 | 0.44 | 1.80 | 8.80 | 0.41 |
|  |  | Trunk | 1.09^a^ | 0.05 | –0.26 | –1.16 | 0.70 | 1.40 | 0.21 |
|  |  | Root | 8.70^c^ | 0.54 | 0.52 | –0.63 | 2.11 | 18.64 | 0.47 |
|  | Evergreen coniferous shrubs (n = 95) | Leaf | 19.60^d^ | 0.84 | –0.72 | –0.46 | 12.62 | 24.13 | 0.17 |
|  |  | Branch | 4.39^b^ | 0.24 | –0.18 | 0.70 | 3.05 | 5.55 | 0.17 |
|  |  | Trunk | 1.44^a^ | 0.08 | –0.47 | –0.46 | 1.20 | 1.64 | 0.12 |
|  |  | Root | 8.80^c^ | 1.05 | 0.25 | –1.14 | 2.10 | 16.03 | 0.48 |
| Forest | Evergreen broad–leaved forest (n = 12) | Leaf | 13.81^c^**^†^** | 1.59 | –0.27 | –2.63 | 10.06 | 17.12 | 0.23 |
|  |  | Branch | 3.51^ab^ | 0.90 | 0.24 | –0.69 | 1.50 | 5.70 | 0.41 |
|  |  | Trunk | 1.00^a^ | 0.13 | 0.55 | 1.50 | 0.70 | 1.35 | 0.27 |
|  |  | Root | 4.60^b^ | 1.09 | –0.41 | 1.17 | 1.80 | 7.09 | 0.47 |
|  | Evergreen coniferous forest (n = 156) | Leaf | 17.75^d^ | 0.60 | 0.15 | 0.14 | 10.04 | 27.87 | 0.21 |
|  |  | Branch | 4.03^b^ | 0.19 | –0.16 | –0.42 | 1.70 | 6.24 | 0.29 |
|  |  | Trunk | 1.19^a^ | 0.06 | 0.35 | –0.19 | 0.60 | 1.98 | 0.29 |
|  |  | Root | 5.55^c^ | 0.39 | 0.75 | 0.03 | 1.20 | 11.77 | 0.46 |
|  | Coniferous and broad–leaved mixed forest (n = 55) | Leaf | 18.81^d^ | 1.45 | –0.78 | 0.19 | 8.25 | 25.62 | 0.27 |
|  |  | Branch | 4.04^b^ | 0.33 | 0.32 | –1.08 | 2.25 | 6.20 | 0.30 |
|  |  | Trunk | 1.21^a^ | 0.08 | 0.33 | –0.72 | 0.80 | 1.70 | 0.24 |
|  |  | Root | 6.44^c^ | 0.90 | 1.36 | 1.81 | 2.15 | 14.92 | 0.54 |

**^†^** Different letters indicate significant differences at the *p* = 0.05 level.

**Table S3** Changes in the nitrogen content (N, mg g–1) of different plant organs among different eco–geographical areas in Tibet Plateau

| **Organs** | **Temperature zone** | **Climate zone** | **N** | **Mean (mg g^–1^)** | **SE** | **Min (g kg^–1^)** | **Max (g kg^–1^)** | **CV** | **Skewness** | **Kurtosis** |
| --- | --- | --- | --- | --- | --- | --- | --- | --- | --- | --- |
| **Leaf** | Total |  | 1564 | 19.21 | 0.67 | 5.59 | 32.89 | 0.24 | –0.02 | 0.18 |
|  | Subfrigid zone | Semi–arid area | 400 | 19.46^c^**^†^** | 0.39 | 9.07 | 32.38 | 0.23 |  |  |
|  |  | Semi–humid area | 264 | 20.00^c^ | 0.37 | 10.73 | 28.37 | 0.17 |  |  |
|  |  | Arid area | 12 | 14.98^a^ | 1.83 | 11.12 | 18.43 | 0.24 |  |  |
|  | Temperate zone | Semi–arid area | 399 | 20.04^c^ | 0.44 | 5.73 | 32.89 | 0.25 |  |  |
|  |  | Semi–humid area | 252 | 18.51^bc^ | 0.42 | 8.25 | 27.87 | 0.21 |  |  |
|  |  | Arid area | 147 | 18.61^bc^ | 0.77 | 5.59 | 32.00 | 0.29 |  |  |
|  |  | Humid area | 6 | 16.23^ab^ | 0.20 | 11.23 | 18.01 | 0.25 |  |  |
|  | Subtropical zone | Humid area | 84 | 15.73^ab^ | 0.93 | 10.04 | 28.49 | 0.31 |  |  |
| **Branch** | Total |  | 584 | 4.11 | 0.32 | 1.50 | 8.80 | 0.35 | 0.55 | 0.55 |
|  | Subfrigid zone | Semi–arid area | 5 | 4.55^b^ | 0.31 | 4.55 | 4.55 | 0.32 |  |  |
|  |  | Semi–humid area | 65 | 4.20^ab^ | 0.35 | 2.80 | 6.80 | 0.30 |  |  |
|  | Temperate zone | Semi–arid area | 45 | 4.85^b^ | 0.63 | 2.20 | 8.60 | 0.39 |  |  |
|  |  | Semi–humid area | 265 | 4.34^b^ | 0.17 | 1.70 | 8.80 | 0.29 |  |  |
|  |  | Arid area | 10 | 4.65^b^ | 0.14 | 4.33 | 4.97 | 0.31 |  |  |
|  | Subtropical zone | Humid area | 130 | 3.27^a^ | 0.29 | 1.50 | 6.33 | 0.45 |  |  |
| **Trunk** | Total |  | 570 | 1.14 | 0.07 | 0.50 | 1.98 | 0.29 | 0.30 | –0.35 |
|  | Subfrigid zone | Semi–arid area | 3 | 1.10a | 0.07 | 1.10 | 1.10 | 0.20 |  |  |
|  |  | Semi–humid area | 27 | 1.35^b^ | 0.07 | 1.16 | 1.80 | 0.27 |  |  |
|  | Temperate zone | Semi–arid area | 18 | 1.00^a^ | 0.15 | 0.70 | 1.70 | 0.30 |  |  |
|  |  | Semi humid area | 138 | 1.20^ab^ | 0.04 | 0.70 | 1.98 | 0.27 |  |  |
|  |  | Arid area | 6 | 1.28^ab^ | 0.04 | 1.20 | 1.37 | 0.25 |  |  |
|  | Subtropical zone | Humid area | 66 | 0.96^a^ | 0.07 | 0.50 | 1.68 | 0.34 |  |  |
| **Root** | Total |  | 1564 | 10.86 | 0.68 | 1.10 | 24.06 | 0.40 | 0.11 | –0.40 |
|  | Subfrigid zone | Semi–arid area | 400 | 12.65^c^ | 0.34 | 4.78 | 22.20 | 0.31 |  |  |
|  |  | Semi–humid area | 264 | 10.26^b^ | 0.41 | 2.11 | 22.49 | 0.37 |  |  |
|  |  | Arid area | 12 | 17.45^d^ | 2.80 | 10.56 | 24.06 | 0.32 |  |  |
|  | Temperate zone | Semi–arid area | 399 | 11.96^bc^ | 0.29 | 2.60 | 20.36 | 0.28 |  |  |
|  |  | Semi–humid area | 252 | 7.05^a^ | 0.34 | 1.20 | 15.34 | 0.44 |  |  |
|  |  | Arid area | 147 | 13.78^c^ | 0.54 | 6.43 | 22.82 | 0.27 |  |  |
|  |  | Humid area | 6 | 6.09^a^ | 0.19 | 5.96 | 6.21 | 0.22 |  |  |
|  | Subtropical zone | Humid area | 84 | 4.91^a^ | 0.50 | 1.10 | 11.61 | 0.49 |  |  |

**^†^** Different letters indicate significant differences at the *p* = 0.05 level.

**Table S4** The results of nitrogen allocation among different organs in natural communities by standardized major axis (SMA) regressions

| Organs  (x vs. y) | Ecosystems | n | *R*^2†^ | *p* | Slope | 95%CI | Intercept | 95%CI | Sig. ‡ |
| --- | --- | --- | --- | --- | --- | --- | --- | --- | --- |
| leaf VS. root | forest | 114 | 0.21 | < 0.001 | 0.65 | (0.55, 0.77) | –5.83 | (–7.92, –3.75) | *** |
|  | grassland | 367 | 0.01 | 0.107 | 0.80 | (0.72, 0.88) | –3.28 | (–4.90, –1.61) | *** |
|  | desert | 22 | 0.53 | < 0.001 | 0.94 | (0.69, 1.29) | –3.34 | (–9.03, 2.34) | ns |
| leaf VS. branch | forest | 98 | 0.52 | < 0.001 | 0.30 | (0.20, 0.35) | –1.54 | (–2.36, –0.72) | *** |
| leaf VS. trunk | forest | 80 | 0.11 | 0.002 | 0.07 | (0.06, 0.09) | –0.12 | (–0.4, 0.15) | *** |
| branch VS. trunk | forest | 84 | 0.14 | < 0.001 | 0.26 | (0.21, 0.32) | 0.15 | (–0.07, 0.36) | *** |
| branch VS. root | forest | 101 | 0.22 | < 0.001 | 1.69 | (1.42, 2.01) | –1.47 | (–2.79, –0.16) | *** |
| trunk VS. root | forest | 85 | 0.09 | 0.006 | 6.48 | (5.27, 7.97) | –2.18 | (–3.81,–0.55) | *** |

^†^ *R*^2^, the coefficient of determination; CI, confidence interval.

^‡^ ***, denote significant differences between the slope of equation and 1 at *p* = 0.001; ns, no significant difference between the slope of equation and 1 at *p* = 0.05.

^§^ The nitrogen contents of all plant organs were transformed into a log–log scale. The likelihood ratio test was used to examine differences between organs.

**
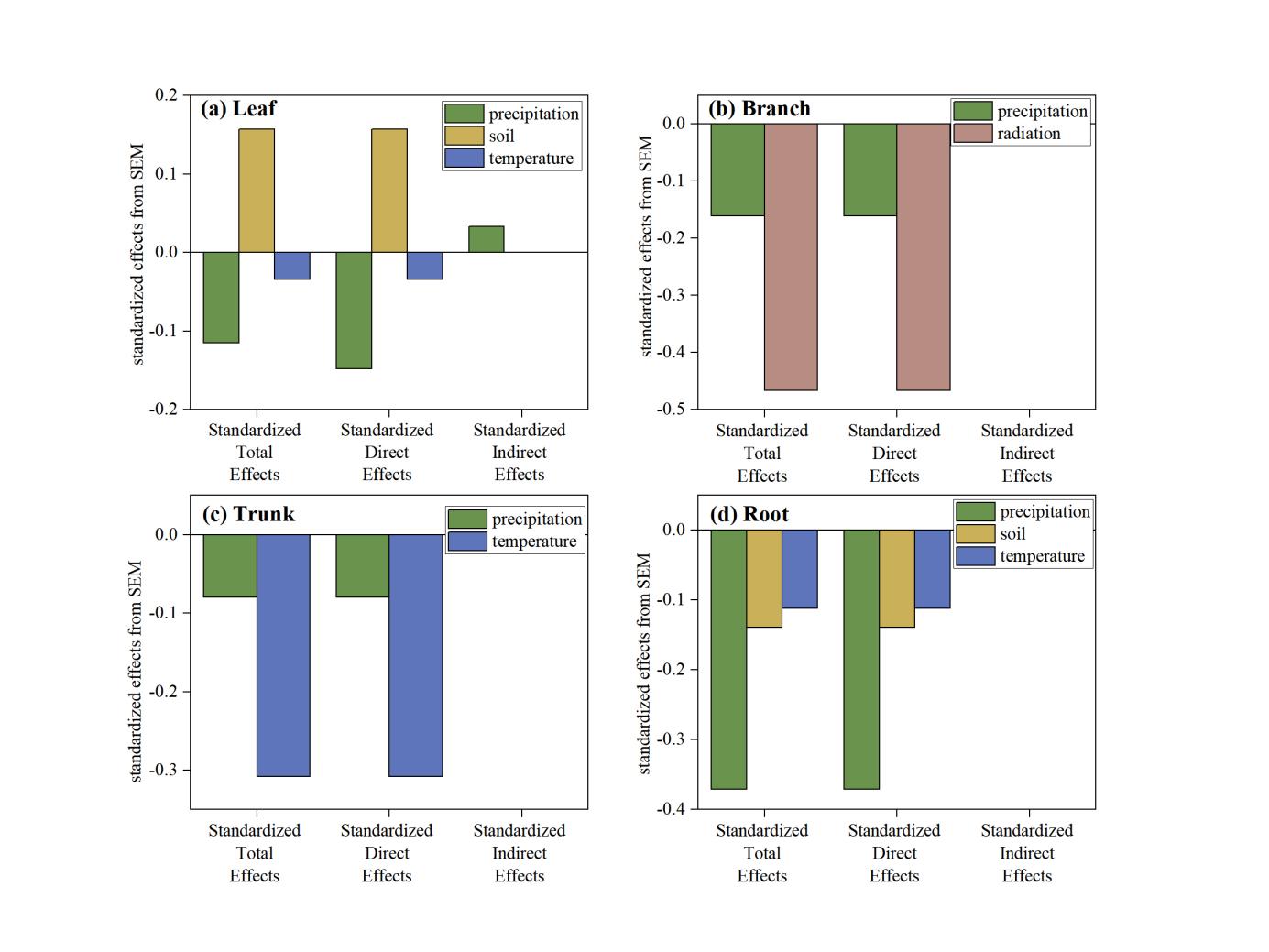
**

**Fig. S1 Path analysis e reveals the effects of environment factors on nitrogen contents (mg g^–1^).** Nitrogen content in different organs was affected by different environmental factors.


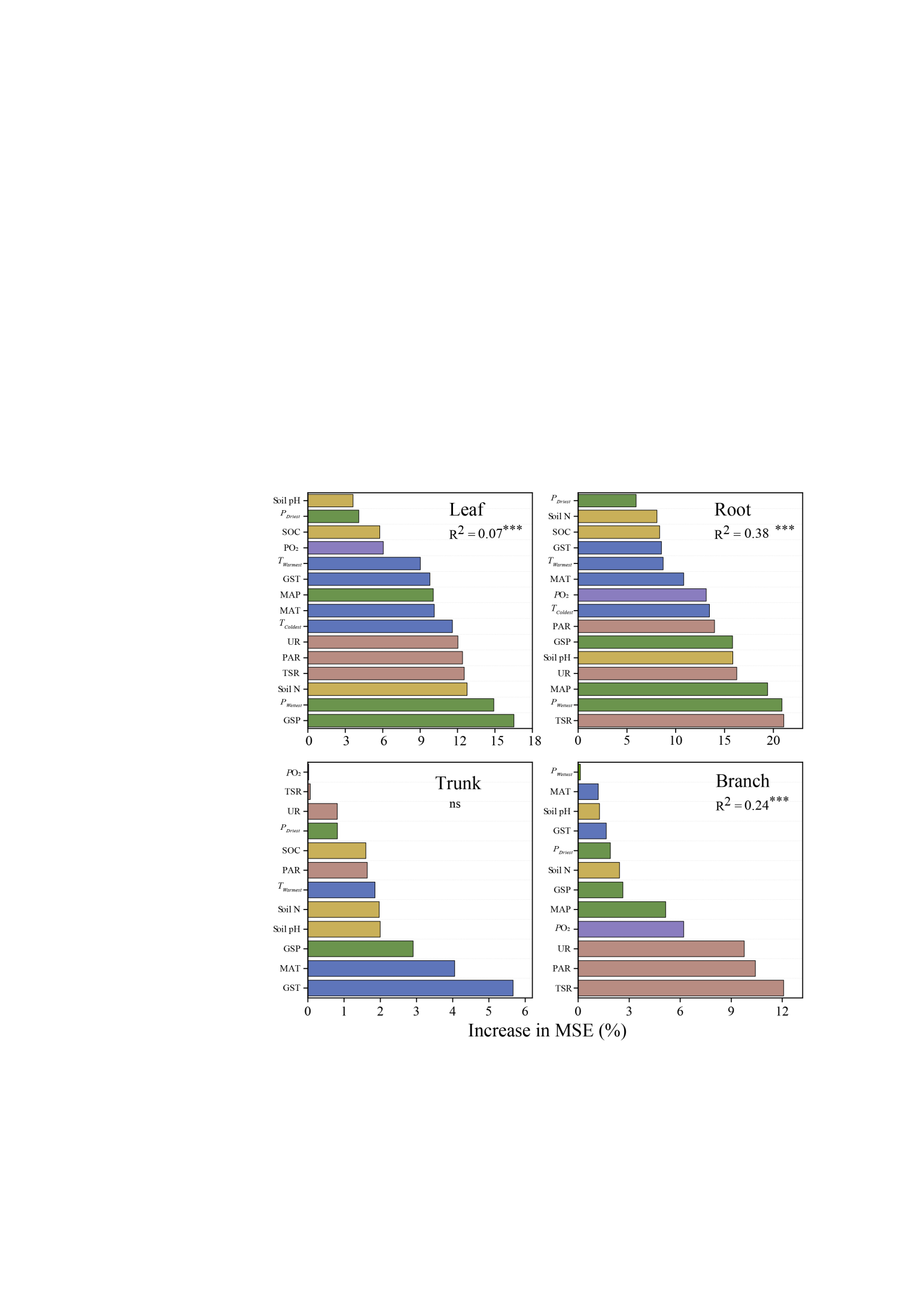


**Fig. S2 Random Forest model reveals environmental controls on the nitrogen contents of different organs.** The nitrogen contents in each organ is affected by multiple environmental factors, but the relative importance of each factor is less than 0.40, and the overall interpretation degree of the model is low. R^2,^ the coefficient of determination; ***, the significance level at *p* = 0.05 level

**
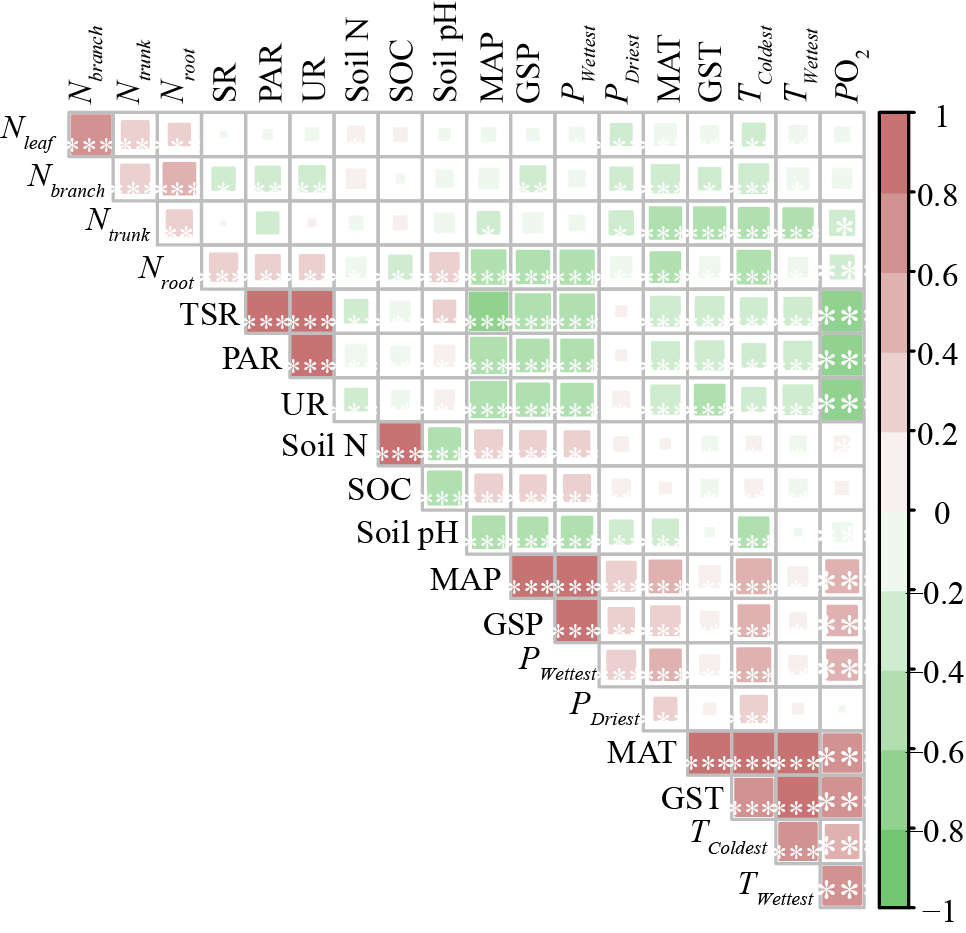
**

**Fig. S3 Correlation analysis between the nitrogen content (N, mg g^–1^) of different organs and environmental factors.**  *, **, ***, *p* < 0.001; 0.01; 0.05. TSR: total solar radiation (mol m^–2^ d^–1^); PAR: photosynthetic active radiation (mol m^–2^ d^–1^); UR: ultraviolet radiation (MJ m^–2^ d^–1^); SOC: soil organic carbon (%); MAP: annual precipitation (mm); GSP: growing season precipitation (mm); *P*_W_: _w_ettest month precipitation (mm); *P*_D_; driest month precipitation (mm); MAT: annual mean temperature (℃); GST: growing season mean temperature (℃); *T*_C_: coldest month mean temperature (℃): *T*_W: w_armest month mean temperature (℃).

**

**

**Fig. S4. Nitrogen homoeostatic regulation coefficient (H) in different organs.** In the community level, the absolute value of H of *N_leaf_,* *N_branch_*, *N_trunk_*, *N_root_* all greater than 1.
